# Anomalous origin of the right pulmonary artery from the ascending aorta: a case report and review of the literature

**DOI:** 10.1097/RC9.0000000000000526

**Published:** 2026-05-14

**Authors:** Alwaleed Al-Dairy, Ali Deeb, Zakaria Aldammad, Ahmad Al-Bitar

**Affiliations:** a Cardiac Surgery Department, Faculty of Medicine, Damascus University, Damascus, Syrian Arab Republic; b Surgery Department, Faculty of Medicine, Damascus University, Damascus, Syrian Arab Republic; c Faculty of Medicine, Damascus University, Damascus, Syrian Arab Republic

**Keywords:** anomalous pulmonary artery, ascending aorta, case report, hemitruncus arteriosus, pediatric cardiac surgery, right pulmonary artery

## Abstract

**Introduction:**

Anomalous aortic origin of a pulmonary artery (AORPA), or hemitruncus arteriosus, is a rare congenital anomaly in which one pulmonary artery arises directly from the ascending aorta. Without timely repair, exposure to systemic pressure leads to irreversible pulmonary vascular disease and high early mortality.

**Case presentation:**

A 4-month-old female infant presented with failure to thrive, tachypnea, and feeding difficulties. Transthoracic echocardiography and CT angiography confirmed an anomalous right pulmonary artery (RPA) originating from the posterior ascending aorta at the sinotubular junction, with a coexisting patent ductus arteriosus (PDA) and elevated right ventricular pressure. Z-scores for the RPA were not calculated because the vessel was already dilated under systemic pressure. She underwent successful direct end-to-side reimplantation of the RPA into the main pulmonary artery, with ligation of the PDA. The postoperative course was uneventful, and she was discharged on day 5 with improved symptoms.

**Clinical Discussion:**

Early surgical correction is critical to prevent pulmonary hypertension and right ventricular overload. Direct tension-free reimplantation is the preferred technique, allowing physiological repair without conduits or patches. Intraoperative assessment confirmed anastomotic patency without residual gradient. Follow-up at 30 days demonstrated a widely patent anastomosis, normalized right ventricular pressure, and satisfactory clinical progress. Structured surveillance is recommended to monitor for restenosis or asymmetric pulmonary artery growth.

**Conclusion:**

Prompt diagnosis and surgical repair of AORPA can restore normal pulmonary circulation and prevent irreversible vascular changes. Even in resource-limited settings, careful imaging and meticulous surgical technique yield excellent early outcomes. Long-term follow-up remains essential.

## Introduction

Anomalous aortic origin of a pulmonary artery (AORPA), or hemitruncus arteriosus, is a rare congenital malformation where one pulmonary artery arises directly from the ascending aorta^[^1–6^]^. It most commonly involves the right pulmonary artery (RPA), originating near the sinotubular junction^[^1–3,6^]^. The anomaly results from abnormal truncal partitioning and sixth arch anomalies, leading to systemic-pressure perfusion of the affected lung and, if untreated, rapid pulmonary vascular remodeling and right ventricular overload^[^1–3,5^]^. Infants typically present early with tachypnea, failure to thrive, and signs of right heart volume/pressure load, often with an associated patent ductus arteriosus (PDA)^[^1–3^]^.

HIGHLIGHTS

- Anomalous origin of a pulmonary artery from the aorta is a rare but life-threatening congenital anomaly that requires prompt diagnosis to prevent irreversible pulmonary hypertension.
- Direct, tension-free end-to-side reimplantation is the preferred surgical technique, offering excellent early patency and potential for growth without the need for conduits.
- Long-term structured surveillance is essential after surgical repair to monitor for anastomotic restenosis, differential pulmonary artery growth, and late-onset pulmonary hypertension.

Transthoracic echocardiography (TTE) is the primary diagnostic tool, with cross-sectional CT or MRI angiography critical for definitive anatomical delineation and operative planning^[^1,2^]^. Accurate imaging is necessary to distinguish AORPA from mimics like aortopulmonary window or truncus arteriosus by identifying two separate semilunar valves, an intact aortic wall, and a normally originating contralateral pulmonary artery^[^1,3,7^]^. Without surgical correction, the natural history is poor, with high early mortality from progressive pulmonary vascular disease, mandating prompt repair to restore normal physiology^[^2,3,5–7^]^.

The preferred surgical strategy is direct, tension-free end-to-side reimplantation of the anomalous artery into the main pulmonary artery (MPA), which offers excellent early patency and growth potential without conduits^[^7–10^]^. Given its rarity, diagnostic challenges, and time-sensitive nature, detailed case reports remain valuable for illustrating successful management pathways^[^1–3,7,10^]^.

While several AORPA cases have been reported, few describe the combination of (1) direct reimplantation performed in a resource-limited setting, (2) the intraoperative use of a simple pressure-based anastomotic adequacy test, and (3) detailed early follow-up with structured surveillance. This case adds value by demonstrating that excellent outcomes are achievable even without advanced intraoperative imaging when meticulous technique and practical intraoperative assessment are employed.

This case report has been reported in line with the SCARE checklist^[11]^.

## Case presentation

A 4-month-old female infant, weighing 4.75 kg and measuring 58 cm in length (both below the 5th percentile for age), was referred for evaluation of failure to thrive, tachypnea, and feeding difficulties. The child had no known syndromic features, cyanosis, or dysmorphic stigmata. Past medical and family histories were unremarkable. No prenatal ultrasound or fetal echocardiography had been performed because routine anomaly scanning was not available in this resource-limited setting.

Physical examination revealed tachypnea, mild subcostal retractions, and hepatomegaly. A systolic murmur was audible at the left upper sternal border. Peripheral pulses were symmetric.

Baseline TTE provided the first definitive clue to the diagnosis. Parasternal short-axis and suprasternal views demonstrated an anomalous RPA originating from the posterior aspect of the ascending aorta, arising close to the sinotubular junction. The vessel measured 7–8 mm in diameter. Z-scores for the RPA diameter were not calculated because the vessel was already dilated under systemic pressure, and normative z-score equations in this age group assume a normal pressure environment; moreover, the software was not routinely available in our setting. The left pulmonary artery (LPA) arose normally from the MPA. The pulmonary valve was tricuspid, morphologically normal, and without stenosis or regurgitation. The MPA was of normal caliber. A PDA was identified, supplying the LPA. Right ventricular systolic pressure was estimated at approximately 70 mmHg, derived from the tricuspid regurgitation jet velocity using an assumed right atrial pressure of 5 mmHg. Right atrial and right ventricular dilation were noted, while left ventricular systolic function remained preserved (fractional shortening and ejection fraction within normal range). Tissue Doppler-derived tricuspid annular plane systolic excursion (TAPSE) and right ventricular fractional area change could not be reliably measured due to suboptimal acoustic windows. No ventricular septal defect (VSD), atrial septal defect, or aortopulmonary window was observed. Systemic venous connections were normal. The overall findings were strongly suggestive of AORPA, prompting further evaluation.

**Figure 1.**
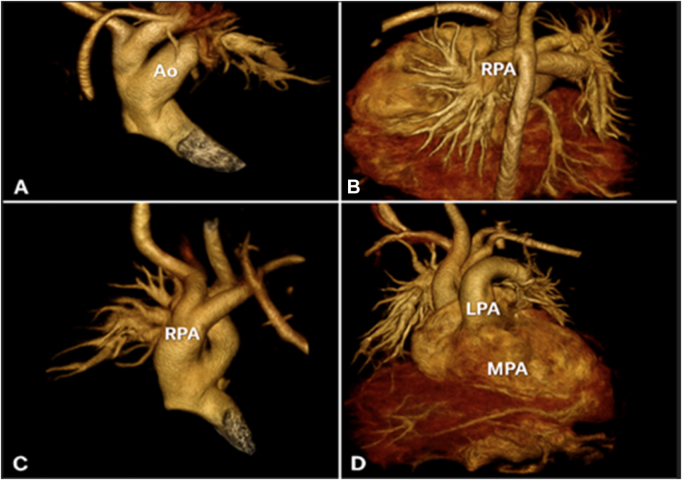
Preoperative 3D volume-rendered CT angiography demonstrating an anomalous origin of the right pulmonary artery (RPA) from the ascending aorta. (A, C) Oblique views showing the RPA arising separately from the aorta, coursing toward the right lung. (B, D) Additional views highlighting the spatial relationship between the aortic origin of the RPA and the normally arising left pulmonary artery (LPA) from the main pulmonary artery (MPA).

CT angiography confirmed the anomalous origin of the RPA from the ascending aorta at the sinotubular junction. The RPA measured 9 mm in diameter, coursing laterally toward the right hilum without extrinsic compression. The LPA arose normally from the MPA. A PDA measuring 3.5 mm connected the descending aorta to the LPA. Three-dimensional reconstructions clearly delineated the spatial separation of the RPA and LPA (Fig. 1A–D).

**Figure 2.**
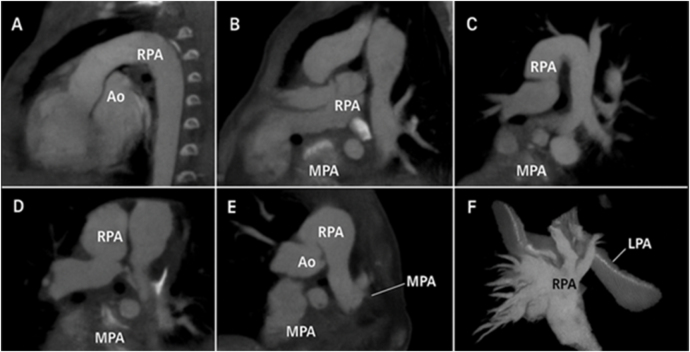
Axial/sagittal reconstruction + 3D volume rendering; arrows indicating the RPA origin at the sinotubular junction; separate panel for the PDA.

Differential diagnoses included truncus arteriosus, aortopulmonary window, or complex VSD with large PDA, which were excluded by the absence of a ventricular septal defect and separate origins of the branch pulmonary arteries (Fig. 2A–F). Symptoms had been noted from early infancy (tachypnea, poor weight gain). After the initial clinical evaluation, echocardiography suggested the anomaly, which was subsequently confirmed by CT angiography. The patient was referred for surgery within days of the diagnosis.

**Figure 3.**
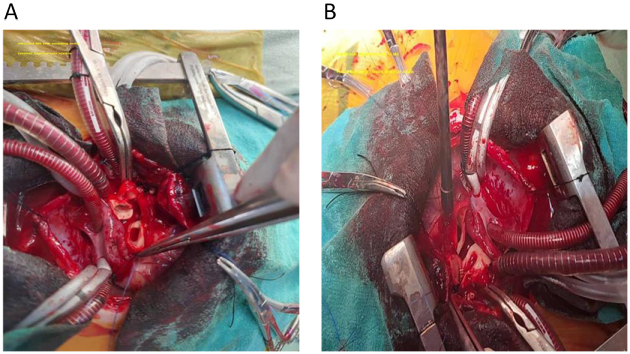
(Intra-op): (A) mobilized RPA stump at the aorta; (B) RPA Distal.

The patient underwent median sternotomy with standard cardiopulmonary bypass (CPB) using aortic and bicaval cannulation. CPB time was 112 minutes, and aortic cross-clamp time was 58 minutes. Myocardial protection was provided with cold blood cardioplegia (St. Thomas solution) delivered antegrade. Intraoperatively, the RPA was identified arising from the ascending aorta at the sinotubular junction. The vessel was carefully mobilized for approximately 15 mm to achieve a tension-free reach to the MPA, taking care to preserve the vasa vasorum and avoid injury to surrounding structures. The RPA was divided flush with the aortic wall, and the aortic defect was closed primarily with a running 7-0 polypropylene suture. The RPA was then anastomosed to the rightward aspect of the MPA using a tension-free end-to-side anastomosis with continuous 7-0 polypropylene suture. To confirm adequate repair, the MPA was briefly occluded proximally while the distal RPA was pressurized; no residual gradient was detected by direct needle pressure measurement (distal RPA pressure = 22 mmHg, simultaneous aortic pressure = 68 mmHg). The PDA was ligated and divided. The patient was weaned from CPB without difficulty on low-dose inotropic support (dopamine 3 µg/kg/min) (Fig. 3).

The infant was transferred to the intensive care unit. No pulmonary hypertensive crises occurred.

Early postoperative echocardiography showed unobstructed flow across the RPA anastomosis, with a peak Doppler gradient of 1.8 m/s and preserved biventricular function. The patient was discharged on postoperative day 5, feeding well, with improved weight gain and normal oxygen saturation in room air.

At the 30-day follow-up, echocardiography demonstrated a patent RPA anastomosis with a normal peak gradient of 2.0 m**/**s. Branch pulmonary artery diameters were normal. Right ventricular size and function were normal. The patient had gained weight appropriately and showed an improved growth trajectory.

Structured surveillance includes echocardiography at 1, 3, 6, and 12 months, then annually, with attention to RPA Doppler peak velocity (intervention threshold **>**2.5–3.0 m**/s**), branch artery z-scores, and right ventricular function. Low-dose aspirin (5 mg/kg/day) is prescribed for the first 6 months postoperatively. Cross-sectional imaging is reserved for cases with elevated gradients or inadequate acoustic windows.

## Discussion

We report a 4-month-old infant with an anomalous origin of the RPA from the ascending aorta (AORPA) who underwent successful direct end-to-side reimplantation of the RPA into the MPA. Early postoperative echocardiography demonstrated unobstructed flow, near-normalization of right ventricular pressures, and satisfactory biventricular function. At 30 days, the patient continued to thrive, with echocardiographic evidence of a widely patent anastomosis and no signs of pulmonary hypertension.

AORPA is a rare anomaly, more frequently involving the RPA, with most reported cases presenting in early infancy due to tachypnea, recurrent infections, or failure to thrive^[^1^**–**^3,6,9^]^. Our patient’s clinical presentation and imaging findings align with these classical features. Previous literature underscores the poor natural history without repair – historical series describe very high early mortality, with most untreated infants succumbing within the first year of life^[^2,3,5,7^]^. Early surgical correction remains the cornerstone of management, and successful reimplantation in infancy is associated with favorable outcomes and preserved pulmonary vascular physiology^[^5^**–**^7,10^]^. In a series by Wang *et al*, direct reimplantation achieved 100% early survival, but restenosis occurred in 12% of cases, highlighting the need for structured surveillance^[^2^]^. Similarly, Jiang *et al* identified young age (<6 months) and preoperative pulmonary hypertension as risk factors for restenosis, reinforcing the importance of timely repair^[^10^]^.

Our experience supports direct, tension-free end-to-side reimplantation as the optimal strategy when mobilization permits favorable geometry. Adequate RPA mobilization (here, ~ 15 mm) avoids kinking. Direct reimplantation minimizes late obstruction, lack of growth potential, and reintervention versus patch or conduit alternatives^[^5^**–**^7^]^. This parallels anomalous left main coronary artery reimplantation, where tension-free mobilization restores flow and long-term patency^[^11^]^. Temporarily occluding the MPA and measuring distal RPA pressure – a simple, resource-appropriate test – confirms anastomotic adequacy, which is especially useful when intraoperative transesophageal echocardiography is unavailable. In resource-limited settings (no prenatal anomaly scanning, z-score software, or TEE), careful preoperative CT angiography and a pressure check enabled success. Such environments require team coordination, low-cost intraoperative tools, and feasible follow-up^[^12^]^. AORPA repair can be safely performed outside high-volume tertiary centers when basic principles are followed.

Continued surveillance is crucial, as late anastomotic narrowing and differential pulmonary artery growth have been reported, occasionally necessitating catheter or surgical re-intervention^[^6,7^]^. Our structured follow-up protocol includes echocardiography at 1, 3, 6, and 12 months, then annually, with the intervention threshold set at an RPA Doppler peak velocity >2.5–3.0 m/s. Low-dose aspirin (5 mg/kg/day) is prescribed for 6 months postoperatively to reduce thrombotic risk at the anastomosis, consistent with our institutional practice. Cross-sectional imaging is reserved for cases with elevated gradients or inadequate acoustic windows.

### Limitations

The follow-up duration is limited to 30 days; longer-term data on anastomotic patency and pulmonary artery growth are not yet available. Additionally, z-scores for branch pulmonary artery diameters could not be calculated due to a lack of normative software in our setting, and TAPSE measurements were suboptimal. Despite these limitations, the early clinical and echocardiographic outcomes are favorable.

## Conclusion

Early recognition and surgical correction of the anomalous origin of the RPA from the ascending aorta are essential to prevent irreversible pulmonary vascular disease. This case highlights that, even in resource-limited settings, careful preoperative imaging, meticulous mobilization, and direct, tension-free reimplantation can restore physiologic pulmonary circulation with excellent early results. Continued follow-up is necessary to monitor anastomotic integrity and branch pulmonary artery growth.

## Data Availability

The data that support the findings of this study are available from the corresponding author upon reasonable request.
